# A Consistent Nonparametric Test for Granger Non-Causality Based on the Transfer Entropy

**DOI:** 10.3390/e22101123

**Published:** 2020-10-03

**Authors:** Cees Diks, Hao Fang

**Affiliations:** 1 Center for Nonlinear Dynamics in Economics and Finance (CeNDEF), University of Amsterdam, Roetersstraat 11, 1018 WB Amsterdam, The Netherlands; H.Fang@uva.nl; 2 Tinbergen Institute, Gustav Mahlerplein 117, 1082 MS Amsterdam, The Netherlands; 3 The UvA Institute for Advanced Study, Oude Turfmarkt 147, 1012 GC Amsterdam, The Netherlands; 4 Accent Groupe, Nieuwezijds Voorburgwal 4-10, 1012 RZ Amsterdam, The Netherlands

**Keywords:** Granger causality, nonparametric test, U-statistic, financial time series, high frequency data, C12, C14, C58, G10

## Abstract

To date, testing for Granger non-causality using kernel density-based nonparametric estimates of the transfer entropy has been hindered by the intractability of the asymptotic distribution of the estimators. We overcome this by shifting from the transfer entropy to its first-order Taylor expansion near the null hypothesis, which is also non-negative and zero if and only if Granger causality is absent. The estimated Taylor expansion can be expressed in terms of a U-statistic, demonstrating asymptotic normality. After studying its size and power properties numerically, the resulting test is illustrated empirically with applications to stock indices and exchange rates.

## 1. Introduction

Characterizing causal interactions between time series has been challenging until Granger in his pioneering work brought forward the concept later known as Granger causality [1]. Since then, testing causal effects has attracted attention not only in Economics and Econometrics, but also in the domains of neuroscience [2,3], biology [4] and physics [5], among others.

The vector autoregressive (VAR) modeling-based test has become a popular methodology over the last decades, with repeated debates on its validity. As we see it, there are at least two critical problems with parametric causality tests. First, being based on a classical linear VAR model, traditional Granger causality tests may overlook significant nonlinear dynamical relationships between variables. As Granger [6] put it, nonlinear models represent the proper way to model the real world which is ‘almost certainly nonlinear’. Secondly, parametric approaches to causality testing bear the risk of model mis-specification. A wrong regression model could lead to a lack of power, or worse, unjustified conclusions. For example, Baek and Brock construct an example where nonlinear causal relations cannot be detected by a traditional linear causality test [7].

In a series of studies, authors have tried to relax parametric model assumptions and provide nonparametric versions of Granger causality tests, which essentially are tests for conditional independence. Hiemstra and Jones were among the first to propose a formal nonparametric approach [8]. By modifying Beak and Brock’s nonparametric method [7], and developing asymptotic theory, Hiemstra and Jones obtained a nonparametric test for Granger causality. However, as this test suffers from a fundamental inconsistency problem, [9] proposed a modified, consistent version of the test based on kernel density estimators, hereafter referred to as the DP test. Alternative semiparametric and nonparametric tests for conditional independence have been proposed based on, among other, additive models [10], the Hellinger distance measure [11], copulas [12], generalized empirical distribution functions [13], empirical likelihood ratios [14] and characteristic functions [15].

The scope of this paper is to provide a novel test for Granger causality, based on the information-theoretical notion of *transfer entropy* (hereafter TE), coined by Schreiber [16]. The transfer entropy was initially used to measure asymmetric information exchange in a bivariate system. By using appropriate conditional densities, the transfer entropy is able to measure information transfer from one variable to another. This property makes it attractive for detecting conditional dependence in dynamical settings in a general (distributional) sense. We refer to [17,18] for detailed reviews of the relation between Granger causality and directed information theory.

Despite the attractive properties of the transfer entropy and related information-theoretical notions, such as the mutual information—the application of concepts from information theory to time series analysis has proved difficult due to the lack of asymptotic theory for nonparametric estimators of these information-theoretical measures. For example, Granger and Lin [19] utilize entropy to detect serial dependence using critical values obtained by simulation. Hong and White [20] prove asymptotic normality for an entropy-based statistic, but the asymptotics only hold for a specific kernel function. Barnett [21] established an asymptotic $\chi^{2}$ distribution for transfer entropy estimators in parametric settings. Establishing asymptotic distribution theory for a fully nonparametric transfer entropy measure is challenging, if not impossible. Diks and Fang [22] provide numerical comparisons to gain some insights into the statistical behavior of nonparametric transfer entropy-based tests.

In this paper, we propose a test statistic based on a first order Taylor expansion of the transfer entropy, which is shown to be asymptotically normally distributed. Instead of deriving the limiting distribution of the transfer entropy—which is hard to track—directly, we bypass the problem by focusing on a quantity that locally (near the null hypothesis) is similar, but globally different, while still sharing the global positive-definiteness property with the transfer entropy. Furthermore, we show that this new test statistic is closely related to the DP test (Section 2.2), and follow a similar approach to finding the asymptotic normal distribution of the estimator of the Taylor expansion.

This paper is organized as follows. Section 2 provides a short introduction to the nonparametric DP test and its lack of power against certain alternatives. Subsequently, the transfer entropy and a nonparametric test based on its first order Taylor expansion near the null hypothesis are introduced. The close linkage of this novel test statistic with the DP test is shown, and asymptotic normality is proved using an asymptotically equivalent U-statistic representation of the estimator. Section 2 also discusses the optimal bandwidth selection rule for specific cases. Section 3 deals with Monte Carlo simulations; three different data generating processes are considered, enabling a direct comparison of size and power between the modified DP test and the DP test. Section 4 considers two financial applications. In the first, we apply the new test to stock volume and return data to make a direct comparison with the DP test; in the second application high frequency exchange rates of main currencies are tested. Finally, Section 5 summarizes.

## 2. A Transfer Entropy-Based Test Statistic for Granger Non-Causality

### 2.1. Nonparametric Granger Non-Causality Tests

This subsection provides some basic concepts and definitions for Granger causality, and the idea of nonparametrically testing for conditional independence. We restrict ourselves to the bivariate setting as it is the most common implementation, although generalization to multivariate densities is possible.

Intuitively, for a strictly stationary bivariate process $\left\{\left(X_{t},Y_{t}\right)\right\}$, t∈Z, it is said that $\left\{X_{t}\right\}$ Granger causes $\left\{Y_{t}\right\}$ if current and past values of $\left\{X_{t}\right\}$ contain some additional information, beyond that in current and past values of $\left\{Y_{t}\right\}$, about future values of $\left\{Y_{t}\right\}$. A linear Granger causality test based on a parametric VAR model can be seen as a special case where testing for conditional independence is equivalent to testing a restriction in the conditional mean specification.

In a more general setting, the null hypothesis of Granger non-causality can be rephrased in terms of conditional dependence between two time series: $\left\{X_{t}\right\}$ is a Granger cause of $\left\{Y_{t}\right\}$ if the distribution of $\left\{Y_{t}\right\}$ conditional on its own history is not the same as that conditional on the histories of both $\left\{X_{t}\right\}$ and $\left\{Y_{t}\right\}$. If we denote the information set of $\left\{X_{t}\right\}$ and $\left\{Y_{t}\right\}$ until time *t* by $F_{Y,t}$ and $F_{X,t}$, respectively, and use ‘∼’ to denote equivalence in distribution, we may give a formal and general definition for Granger causality. For a strictly stationary bivariate process $\left\{\left(X_{t},Y_{t}\right)\right\}$, t∈Z, $\left\{X_{t}\right\}$ is a Granger cause of $\left\{Y_{t}\right\}$ if, for all k∈1,2,…,

$$\left(Y_{t+1},\ldots ,Y_{t+k}\right)\;\left|\;\left(\;F_{Y,t},\;F_{X,t}\;\right)\;≁\;\left(Y_{t+1},\ldots ,Y_{t+k}\right)\;\right|\;F_{Y,t}.$$

In the absence of Granger causality, i.e.,

$$\left(Y_{t+1},\ldots ,Y_{t+k}\right)\;\left|\;\left(\;F_{Y,t},\;F_{X,t}\;\right)\;∼\;\left(Y_{t+1},\ldots ,Y_{t+k}\right)\;\right|\;F_{Y,t},$$

$\left\{X_{t}\right\}$ has no influence on the distribution of future $\left\{Y_{t}\right\}$. This is also referred to as Granger non-causality and often expressed as conditional independence between $\left\{X_{t}\right\}$ and $\left\{Y_{t}\right\}$ as

(1) $$\left(Y_{t+1},\ldots ,Y_{t+k}\right)\;⊥\;\left(X_{t},\ldots ,X_{t-m}\right)\;|\;F_{Y,t},$$

for m=0,1,2,…, Granger non-causality, as expressed in Equation (1), lays the first stone for a nonparametric test without imposing any parametric assumptions, apart from strict stationarity and weak dependence, about the data generating process or underlying distributions for $\left\{X_{t}\right\}$ and $\left\{Y_{t}\right\}$. The orthogonality here concerns not only the conditional mean, but also higher conditional moments. We assume two things here. First, $\left\{\left(X_{t},Y_{t}\right)\right\}$ is a strictly stationary bivariate process. In practice, it is infeasible in nonparametric settings to condition on the entire past of $X_{t}$ and $Y_{t}$. We therefore implicitly consider the process to be of finite Markov orders, $l_{X}<\infty$ and $l_{Y}<\infty$ in the past of $X_{t}$ and $Y_{t}$, respectively.

The null hypothesis of Granger non-causality is that *$H_{0}$: $\left\{X_{t}\right\}$ is not a Granger cause of $\left\{Y_{t}\right\}$*. To keep focus on the main contribution of this paper—the Taylor expansion of the transfer entropy and its asymptotic distribution—in this paper we limit ourselves to the bivariate case with single lags in the past such that $k=l_{X}=l_{Y}=1$, which so far has been the case considered most in the literature on nonparametric Granger non-causality. Extensions to higher lags and/or higher-variate processes are feasible, but require reduction of the bias order by data sharpening or higher-order density estimation kernels (see e.g., Diks and Wolski, 2016). We define the three-variate vector $W_{t}=\left(X_{t},Y_{t},Z_{t}\right)$, where $Z_{t}=Y_{t+1}$; and W=(X,Y,Z) indicates a random variable *W* with distribution equal to the invariant distribution of $W_{t}$. Within the bivariate setting, *W* is a three-dimensional vector. In terms of density functions $f_{\cdot }\left(\cdot \right)$ (which are assumed to exist), and given $k=l_{X}=l_{Y}=1$, Equation (1) can be phrased as

(2) $$H_{0}:\;\frac{f_{X,Y,Z}\left(x,y,z\right)}{f_{Y}\left(y\right)}=\frac{f_{Y,Z}\left(y,z\right)}{f_{Y}\left(y\right)}\frac{f_{X,Y}\left(x,y\right)}{f_{Y}\left(y\right)},$$

for all (x,y,z) in the support of *W*, or equivalently as

(3) $$H_{0}:\;\frac{f_{X,Y,Z}\left(x,y,z\right)}{f_{Y}\left(y\right)}-\frac{f_{Y,Z}\left(y,z\right)}{f_{Y}\left(y\right)}\frac{f_{X,Y}\left(x,y\right)}{f_{Y}\left(y\right)}=0,$$

for all (x,y,z) in the support of *W*. A nonparametric test for Granger non-causality seeks to find statistical evidence of violation of Equation (2) or Equation (3). There are many nonparametric measures available for this purpose, some of which are mentioned above. However, as far as we know, the DP test, to be described below, currently is the only fully nonparametric test that is known to have correct asymptotic size under the null hypothesis of Granger non-causality.

### 2.2. The DP Test

Hiemstra and Jones [8] proposed to test the condition expressed by Equation (2) by calculating correlation integrals for each density and measuring the discrepancy between two sides of the equation. However, their test is known to suffer from severe size distortion due to the fact that the quantity on which the test is based is inconsistent with Equation (2). To overcome this problem, DP suggest to use a conditional dependence measure by incorporating a local weight function g(x,y,z) and formulating Equation (3) as

(4) $$E\left[\left(\frac{f_{X,Y,Z}\left(X,Y,Z\right)}{f_{Y}\left(Y\right)}-\frac{f_{Y,Z}\left(Y,Z\right)}{f_{Y}\left(Y\right)}\frac{f_{X,Y}\left(X,Y\right)}{f_{Y}\left(Y\right)}\right)g\left(X,Y,Z\right)\right]=0.$$

Under the null hypothesis of no Granger causality, the term within the large round brackets vanishes, and the expectation goes to zero. As noted in [23], Equation (4) can be treated as an infinite number of moment restrictions. Although testing for Equation (4) for a specific function *g* instead of testing Equation (2) or Equation (3) may lead to a loss of power against some specific alternatives, there is also an advantage to do so. For example, in the DP test, the weight function g(x,y,z) is taken to be $g\left(x,y,z\right)=f_{Y}^{2}\left(y\right)$, as this leads to a U-statistic representation of the corresponding estimator, which enables the analytical derivation of the asymptotic normality of the test statistic. In principle, other choices for g(x,y,z) will also do as long as the test has satisfactory power against alternatives of interest. Since in the DP test, $g\left(x,y,z\right)=f_{Y}^{2}\left(y\right)$, it tests the implication

(5) $$H_{0}^{\prime }:\;q\equiv E\left[f_{X,Y,Z}\left(X,Y,Z\right)f_{Y}\left(Y\right)-f_{X,Y}\left(X,Y\right)f_{Y,Z}\left(Y,Z\right)\right]=0,$$

of $H_{0}$, rather than $H_{0}$ itself.

Given a local density estimator of a $d_{W}$-variate random vector *W* at $W_{i}$ as

(6) $${\hat{f}}_{W}\left(W_{i}\right)={\left(\left(n-1\right)h\right)}^{-d_{W}}\sum_{j,j\neq i}^{n}K\left(\frac{W_{i}-W_{j}}{h}\right),$$

where K is a finite variance, zero mean, kernel density function (e.g., the standard normal density function) and *h* is the bandwidth, the DP test develops a third order U-statistic estimator for the functional *q*, given by

(7) $$T_{n}\left(h\right)=\frac{\left(n-1\right)}{n(n-2)}\sum_{i}\left({\hat{f}}_{X,Y,Z}\left(X_{i},Y_{i},Z_{i}\right){\hat{f}}_{Y}\left(Y_{i}\right)-{\hat{f}}_{X,Y}\left(X_{i},Y_{i}\right){\hat{f}}_{Y,Z}\left(Y_{i},Z_{i}\right)\right),$$

where the normalization factor (n−1)/(n(n−2)) is inherited from the U-statistic representation of $T_{n}\left(h\right)$. It is worth mentioning that a second order square (or rectangular) kernel K is adopted by DP. However, there are two main drawbacks of using a square kernel. First, a square kernel will yield a discontinuous density estimate $\hat{f}\left(\cdot \right)$, which is not attractive from a practical perspective. Second, it weighs all neighbor points $W_{j}$ equally, overlooking their relative distance to the estimation point $W_{i}$. Therefore, a smooth kernel function—the Gaussian kernel—is used here, namely the product kernel function defined as $K\left(W\right)=\prod_{s=1}^{d_{W}}\kappa \left(w_{s}\right)$, where $w_{s}$ is $s^{\mathrm{th}}$ element in *W*. Using a standard univariate Gaussian kernel, $\kappa \left(w_{s}\right)={\left(2\pi \right)}^{-1/2}e^{-\frac{1}{2}{\left(w_{s}\right)}^{2}}$, K(.) is the standard multivariate Gaussian kernel as described in [24,25].

For $l_{X}=l_{Y}=1$, DP prove the asymptotic normality of $T_{n}\left(h\right)$. Namely, if the bandwidth $h=h_{n}$ depends on the sample size as $h_{n}=Cn^{-\beta }$ for constants C>0 and $\beta \in (\frac{1}{4},\frac{1}{3})$, then the test statistic in Equation (7) satisfies

(8) $$\begin{array}{c} \sqrt{n}\frac{T_{n}\left(h\right)-q}{S_{n}}\overset{d}{⟶}N\left(0,1\right), \end{array}$$

where $S_{n}^{2}$ is a consistent estimator of the asymptotic variance of $T_{n}\left(h\right)$. DP suggest to implement an one-sided version of the test, rejecting $H_{0}^{\prime }$: q=0 against the alternative $H_{a}$: q>0 if $T_{n}\left(h\right)$ is too large. That is, given the asymptotic critical value $z_{1-\alpha }$, the null hypothesis $H_{0}^{\prime }$ is rejected at significance level α if $\sqrt{n}T_{n}\left(h\right)/S_{n}>z_{1-\alpha }$.

### 2.3. Inconsistency of the DP Test

The drawback of the DP test arises from the fact that $H_{0}^{\prime }$ in Equation (5), obtained for a specific weight function g(x,y,z), need not be equivalent to $H_{0}$ in Equations (2) and (3); it merely is an implication of $H_{0}$. For consistently testing $H_{0}$, an analogue of *q* is desirable that satisfies the positive definiteness property stated next, which *q* does not satisfy.

**Definition** **1.** *A functional s of the distribution of W is positive definite if s≥0 with s=0 if and only if $X_{t}$ and $Z_{t}$ are conditionally independent given $Y_{t}$.*

From the previous reasoning, it is obvious that Equation (5) is implied by Equation (3), and Definition 1 states that a strictly positive *q* is achieved if and only if $H_{0}$ is violated. In other words, the null hypothesis of Granger non-causality requires that $X_{t}$ and $Z_{t}$ are independent conditionally on $Y_{t}$, which is just a sufficient, but not a necessary, condition for q=0. With Definition 1, $H_{0}^{\prime }$ coincides with $H_{0}$ and a consistent estimator of *q*, i.e., $T_{n}\left(h\right)$ as suggested by DP, will have unit asymptotic power. If this property is not satisfied, a test for q=0 could deviate from the test on $H_{0}$. Although [23] identified specific sub-classes of processes for which *q* is positive definite, we can easily construct a counterexample where the DP test has no power even if $X_{t}$ strongly Granger causes $Z_{t}$. For completeness, such a counterexample is given next.

Inspired by the example in [26], where a closely-related test for unconditional independence is proposed, we consider a conditional counterpart to illustrate that *q* is not positive definite. The one-sided DP test will be seen to suffer from a lack of power for this example process. As we show below, in the case where q=0, this drawback cannot be overcome even with a two-sided DP test.

Consider the process $\left\{\left(X_{t},Y_{t},Z_{t}\right)\right\}$ where, as before, $Z_{t}\equiv Y_{t+1}$. We assume that the i.i.d. continuous variable $X_{t}\in \left[-1,1\right]$, with probability 1−d of being positive, where 0<d<1. Further, there is no dependence between $X_{t}$ and $Y_{t}$, and $Z_{t}$ does not depend on $Y_{t}$ but on $X_{t}$ in such a way that the conditional density of $\left(X_{t},Z_{t}|Y_{t}=y\right)$ is given by

(9) $$f\left(x_{t},z_{t}|y_{t}\right)=f\left(x_{t},z_{t}\right)=\left\{\begin{array}{cc} 1-2d, & \mathrm{if}\;0\leq x_{t}\leq 1,\;0\leq z_{t}\leq 1,\\ d, & \mathrm{if}\;0\leq x_{t}\leq 1,\;-1\leq z_{t}<0,\\ d, & \mathrm{if}\;-1\leq x_{t}<0,\;0\leq z_{t}\leq 1,\\ 0, & \mathrm{if}\;-1\leq x_{t}<0,\;-1\leq z_{t}<0, \end{array}\right.$$

for $0\leq d\leq \frac{1}{2}$.

Given Equation (9), the marginal densities of $X_{t}$, $Y_{t}$ and $Z_{t}$ can be calculated to be all equal, with $P(-1\leq X_{t}<0)=d$ and $P(0\leq X_{t}\leq 1)=1-d$, while the conditional probability of $Z_{t}$ being larger than zero given $\left\{\left(X_{t}=x_{t},Y_{t}=y_{t}\right)\right\}$ is given by $P(0<Z_{t}\leq 1|\left(x_{t},y_{t}\right))=1$ for $-1\leq x_{t}<0$, and $P\left(0\leq Z_{t}\leq 1|\left(x_{t},y_{t}\right)\right)=\left(1-2d\right)/\left(1-d\right)$ for $0\leq x_{t}\leq 1$. Hence, for $0<d\leq \frac{1}{2}$, $\left\{X_{t}\right\}$ is a Granger cause of $\left\{Y_{t}\right\}$ since $X_{t}$ has an impact on the distribution of $Z_{t}=Y_{t+1}$, given $Y_{t}$. For this example, we can explicitly calculate *q* defined in Equation (5), which is found to be $q=d^{2}\left({\left(1-d\right)}^{3}+d^{3}\right)\left(4d-1\right)$. For $0<d<\frac{1}{4}$, *q* has a negative value. In this situation, the one-sided DP test, which rejects for large *q*, is not a consistent test for Granger non-causality. One may argue that this is not a problem if we use a two-sided test at the price of losing some power. However, the inconsistency of the DP test—which tests $H_{0}^{\prime }$ rather than $H_{0}$—then would still be illustrated by the example if $d=\frac{1}{4}$, for which q=0 exactly, while $\left\{X_{t}\right\}$ is clearly a Granger cause of $\left\{Y_{t}\right\}$; the DP test will only have trivial power against this alternative.

**Figure 1:**
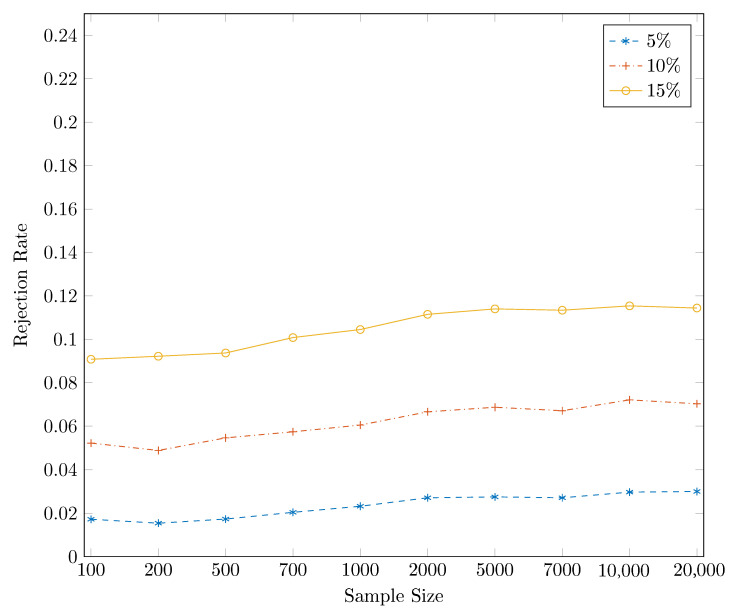
Power for the one-sided DP test for the artificial process $\left\{\left(X_{t},Y_{t},Z_{t}\right)\right\}$ for d=1/4, for which q=0, at nominal size, from the bottom to the top, 5%, 10% and 15%, respectively, based on 10,000 independent simulations.

**Figure 2:**
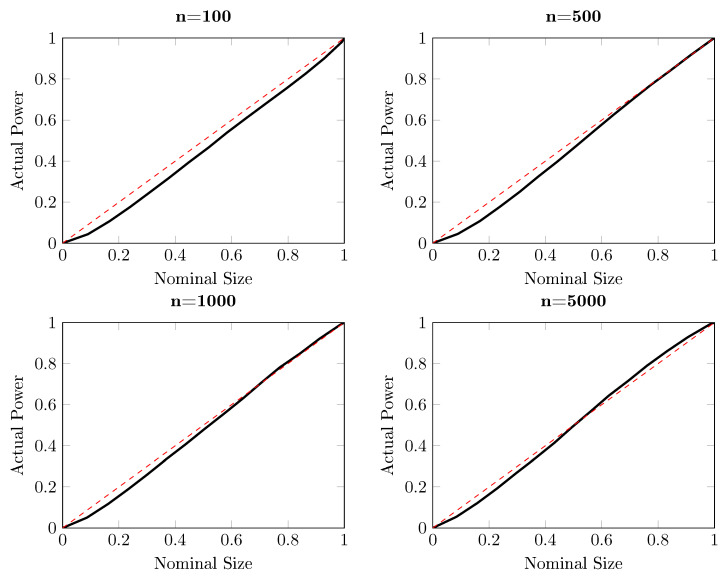
Size-power plots for the one-sided DP test for the artificial process $\left\{\left(X_{t},Y_{t},Z_{t}\right)\right\}$ for d=1/4, for which q=0, based on 10,000 independent simulations. Each subplot draws the actual power against the nominal size for different sample sizes, ranging from 100 to 5000. The solid curve represents the actual power and the red dash line indicates the diagonal, indicating the nominal size of a test.

Figure 1 reports the power of the one-sided DP test as a function of the sample size for different significance levels, based on 10,000 independent simulations. Three nominal sizes are illustrated here: 5%, 10% and 15%, and the sample size ranges from 100 to 20,000. It is striking from Figure 1 that the DP test hardly has power against the alternative with d=1/4, for which q=0. The same conclusion can be drawn from Figure 2, where the size-power plots [27] are given. For almost all sub-panels with different sample sizes, the power of the DP test is around the diagonal line for this particular example when q=0, which indicates that the DP test has only trivial power to detect Granger causality from $X_{t}$ to $Y_{t}$.

The lack of power of the one-sided DP test in this example is hardly alleviated by its two-sided counterpart, as a result of the absence of equivalence between q=0 and conditional independence. The difference between $H_{0}$ and its implication $H_{0}^{\prime }$ gives rise to the lack of power of the DP test as the estimated quantity is not positive definite. In the next subsection, a new test statistic, based on the information-theoretical concept transfer entropy, is introduced and the test statistic is shown to be positive definite, which overcomes the inherited drawback of the DP test. In fact, this new test statistic shares many similarities with the DP test statistic, but also has an information-theoretical interpretation for its non-negativity.

### 2.4. Information-Theoretical Interpretation

In a very different context from testing for conditional independence, the problem of information feedback and impact also has drawn much attention since 1950. Information theory, as a branch of applied mathematical theory of probability and statistics, studies the transmission of information over a noisy channel. This entropy, also referred to as Shannon entropy, is a key measure in the field of information theory brought forward in [28,29]. The entropy measures the uncertainty and randomness associated with a random variable. Suppose that *S* is a random vector with density $f_{S}\left(s\right)$, then the Shannon entropy is defined as

$$H\left(S\right)=-\int f_{S}\left(s\right)\log\left\{f_{S}\left(s\right)\right\}\;ds.$$

There is a long history of applying information measures in econometrics. For example, ref. [30] uses the Kullback–Leibler information criterion (KLIC) [31] to construct a one-sided test for serial independence. Since then, nonparametric tests using entropy-based measures for independence between two time series are becoming prevalent. Granger and Lin [19] use entropy measure to identify the lags in a nonlinear bivariate model. Granger et al. [32] study dependence with a transformed metric entropy, which has the additional advantage of allowing multiple comparisons of distances and turns out to be a proper measure of distance. Hong and White [20] provide a new entropy-based test for serial dependence, and show that the test statistic is asymptotically normal.

Although inspiring, those results cannot be applied directly to measure conditional dependence. We therefore consider that the transfer entropy (TE) introduced in [16] is a suitable measure to serve this purpose. The TE quantifies the amount of information explained in one series *k* steps ahead from the state of another series, given the information contained in its own past. We briefly introduce the TE and KLIC before we further discuss its relation with the modified DP test.

Suppose that we have a bivariate process $\left\{\left(X_{t},Y_{t}\right)\right\}$, and for brevity we put $X=\{X_{t}\}$, $Y=\{Y_{t}\}$ and $Z=\{Y_{t+k}\}$. Again, we limit ourselves to k=1 lag for simplicity, and consider the three-dimensional vector W=(X,Y,Z) as before. The transfer entropy ${\mathrm{TE}}_{X\to Y}$ is a nonlinear and nonparametric measure for the amount of information contained in *X* about *Z*, in addition to the information about *Z* that already contained in *Y*. Although the TE defined in [16] applies to discrete variables, it is easily generalized to continuous variables. Conditional on *Y*, ${\mathrm{TE}}_{X\to Y}$ is defined as

(10) $$\begin{array}{cc} {\mathrm{TE}}_{X\to Y} & =E_{W}\left(\log\frac{f_{Z,X|Y}\left(Z,X|Y\right)}{f_{X|Y}\left(X|Y\right)f_{Z|Y}\left(Z|Y\right)}\right)\\ \; & =\int \int \int f_{X,Y,Z}\left(x,y,z\right)\log\frac{f_{X,Z|Y}\left(x,z|y\right)}{f_{X|Y}\left(x|y\right)f_{Z|Y}\left(z|y\right)}\;dx\;dy\;dz\\ \; & =E_{W}\left(\log\frac{f_{X,Y,Z}\left(X,Y,Z\right)}{f_{Y}\left(Y\right)}-\log\frac{f_{X,Y}\left(X,Y\right)}{f_{Y}\left(Y\right)}-\log\frac{f_{Y,Z}\left(Y,Z\right)}{f_{Y}\left(Y\right)}\right)\\ \; & =E_{W}\left(\log f_{X,Y,Z}\left(X,Y,Z\right)+\log f_{Y}\left(Y\right)-\log f_{X,Y}\left(X,Y\right)-\log f_{Y,Z}\left(Y,Z\right)\right). \end{array}$$

Using the conditional mutual information I(Z,X|Y=y), the TE can be equivalently formulated in terms of four Shannon entropies as

$$\begin{array}{cc} {\mathrm{TE}}_{X\to Y} & =I(Z,X|Y)\\ \; & =H(Z|Y)-H(Z|X,Y)\\ \; & =H(Z,Y)-H(Y)-H(Z,X,Y)+H(X,Y). \end{array}$$

In order to construct a test for Granger causality based on the TE, it remains to be shown that the TE is a proper basis for testing the null hypothesis. The following theorem, as a direct result of the properties of the KLIC, lays the quantitative foundation for testing based on the TE.

**Theorem** **1.** *The transfer entropy ${\mathrm{TE}}_{X\to Y}$, as a functional of the joint density of W=(X,Y,Z), is positive definite; that is, ${\mathrm{TE}}_{X\to Y}\geq 0$ with equality if and only if $f_{Z,X|Y}\left(z,x|y\right)=f_{X|Y}\left(x|y\right)f_{Z|Y}\left(z|y\right)$ for all (x,y,z) in the support of W.*

**Proof.**  Equation (1) follows from generalizing Theorem 3.1 in Chapter 2 of [33], where the divergence between two different densities has been considered. An alternative proof is given in Equation (A.1) in Appendix A by using Jensen’s inequality and concavity of the log function. □

It is not difficult to verify that the condition for ${\mathrm{TE}}_{X\to Y}=0$ coincides with Equations (2) and (3) for Granger non-causality under the null hypothesis. This positive definiteness makes ${\mathrm{TE}}_{X\to Y}$ a desirable measure for constructing a one-sided test of Granger causality; any divergence from zero is a sign of conditional dependence of *Y* on *X*. To estimate ${\mathrm{TE}}_{X\to Y}$, one may follow the recipe in [34] by measuring *k*-nearest neighbor distances. A more natural method, applied in this paper, is to use the plug-in kernel estimates given in Equation (6), and replace unknown expectations by sample averages.

However, the direct use of the TE to test Granger non-causality is not easy due to the lack of asymptotic theory for the test statistic. It has been shown [19] that the asymptotic distribution of entropy-based estimators usually depends on strict assumptions regarding the dataset. Over the years several break-throughs have been made with the application of entropy to testing serial independence, e.g., [30] obtains an asymptotic N(0,1) distribution for an entropy measure by a sample-splitting technique and [20] derives asymptotic normality under bounded support data and quartic kernel assumptions. However, the limiting distribution of the natural nonparametric TE estimator is still unknown under more general conditions.

One may argue in favor of using simulation techniques to overcome the problem of the lack of asymptotic theory. However, as suggested in [11], there exist estimation biases of TE statistics for non-parametric dependence measures under the smoothed bootstrap procedure. Even with parametric test statistics, it has been noticed [21] that the TE-based estimator is generally biased. Surrogate data are also applied widely, for instance in [35,36] to detect information transfer. We therefore consider the direct usage of the TE for nonparametric tests for Granger non-causality difficult, if not impossible.

Below, we show that a first order Taylor expansion of the TE provides a way out to construct the asymptotic distribution of this meaningful information measure. In the next section, we show that the first order Taylor expansion of the TE can form the basis of a modified DP test for conditional independence. This not only helps to circumvent the problem of asymptotic distribution for entropy-based statistic, but also endows the modified DP test with positive definiteness.

In the remaining part of this section we will introduce the first order Taylor expansion of the TE, and the positive definiteness of the measure will be given afterwards. Starting with Equation (10), we perform the first order Taylor expansion locally at ${\mathrm{TE}}_{X\to Y}=0$, which is

(11) $$\begin{array}{cc} {\mathrm{TE}}_{X\to Y} & =E_{W}\left[\log\frac{f_{Z,X|Y}\left(Z,X|Y\right)}{f_{X|Y}\left(X|Y\right)f_{Z|Y}\left(Z|Y\right)}\right]\\ \; & =E_{W}\left[\log\left(1+\left(\frac{f_{Z,X|Y}\left(Z,X|Y\right)}{f_{X|Y}\left(X|Y\right)f_{Z|Y}\left(Z|Y\right)}-1\right)\right)\right]\\ \; & =E_{W}\left[\frac{f_{Z,X|Y}\left(Z,X|Y\right)}{f_{X|Y}\left(X|Y\right)f_{Z|Y}\left(Z|Y\right)}-1\right]+h.o.t., \end{array}$$

where ‘h.o.t’ stands for ‘higher order terms’ in $\frac{f_{Z,X|Y}\left(Z,X|Y\right)}{f_{X|Y}\left(X|Y\right)f_{Z|Y}\left(Z|Y\right)}-1$, which is small close to the null hypothesis. Ignoring the higher order terms in the transfer entropy makes the distribution of the test statistic tractable, without (up to leading order) affecting the dependence measure close to the null hypothesis, where by definition a powerful test is needed.

By ignoring higher order terms, we define the first order expansion $\vartheta =E_{W}\left[\frac{f_{Z,X|Y}\left(Z,X|Y\right)}{f_{X|Y}\left(X|Y\right)f_{Z|Y}\left(Z|Y\right)}-1\right]$ as a measure for conditional dependence. The following theorem states that ϑ, which with slight abuse of language we still refer to as a transfer entropy, inherits the positive definiteness of the TE.

**Theorem** **2.** *The transfer entropy ϑ, as a functional of the joint density of W=(X,Y,Z), is positive definite; that is, ϑ≥0 with equality if and only if $f_{Z,X|Y}\left(z,x|y\right)=f_{X|Y}\left(x|y\right)f_{Z|Y}\left(z|y\right)$ for all (x,y,z) in the support of W.*

**Proof.**  See Equation (A.2). □

Equation (2) indicates that the divergence measure ϑ has the desirable property of positive definiteness, which the measure *q* used in the DP test is lacking. However, direct estimation of Equation (11) does not lead to a practically useful test statistic without the asymptotic distribution. In the next subsection we show that the nonparametric estimator of ϑ is asymptotically normal. The key to this result is the fact that the DP statistic and the newly proposed statistic only differ in terms of the weight function g(x,y,z) in Equation (4), and that the proof of asymptotic normality of the DP test can be easily adjusted to accommodate this new weight function.

### 2.5. A Modified DP Test

In comparing Equations (3) and (4), it can be seen that the discrepancy between $H_{0}^{\prime }$ and $H_{0}$ arises from incorporation of the weight function $g\left(x,y,z\right)=f_{Y}^{2}\left(y\right)$ to the null hypothesis. In principle other positive functions g(x,y,z) can be used, such as those discussed by DP. As long as the corresponding estimator of the divergence measure has a U-statistic representation, asymptotic normality follows from the theory of U-statistics. Particularly, we propose to modify the DP test by dividing all terms in the expectation of Equation (5) by the function $v\left(x,y,z\right)\equiv f_{X,Y}\left(x,y\right)f_{Y,Z}\left(y,z\right)$, since then, by Theorem 2

(12) $$H_{0}^{\prime \prime }:\;E\left[\left(f_{X,Y,Z}\left(X,Y,Z\right)f_{Y}\left(Y\right)-f_{X,Y}\left(X,Y\right)f_{Y,Z}\left(Y,Z\right)\right)\frac{1}{v(X,Y,Z)}\right]=0,$$

is not just implied by $H_{0}$, but equivalent to it.

One can also think of Equation (12) as the result of plugging in a different weight function in Equation (4). By the choice $g\left(x,y,z\right)=f_{Y}^{2}\left(y\right)/\left(f_{X,Y}\left(x,y\right)\times f_{Y,Z}\left(y,z\right)\right)$ instead of $g\left(x,y,z\right)=f_{Y}^{2}\left(y\right)$, which was used by DP, Equation (12) simplifies to

(13) $$H_{0}^{\prime \prime }:\;\vartheta \equiv E\left[\frac{f_{X,Y,Z}\left(X,Y,Z\right)f_{Y}\left(Y\right)}{f_{X,Y}\left(X,Y\right)f_{Y,Z}\left(Y,Z\right)}-1\right]=0,$$

which is equivalent to the first order Taylor expansion in Equation (11) and hence to $H_{0}$ by Equation (2). To estimate ϑ, we propose to use the following statistic with density estimator defined in Equation (6):

(14) $$T_{n}^{\prime }\left(h\right)=\frac{\left(n-1\right)}{n(n-2)}\sum_{i=1}^{n}\left[\left({\hat{f}}_{X,Y,Z}\left(X_{i},Y_{i},Z_{i}\right){\hat{f}}_{Y}\left(Y_{i}\right)-{\hat{f}}_{X,Y}\left(X_{i},Y_{i}\right){\hat{f}}_{Y,Z}\left(Y_{i},Z_{i}\right)\right)\frac{1}{\hat{v}\left(X_{i},Y_{i},Z_{i}\right)}\right],$$

where $\hat{v}\left(X_{i},Y_{i},Z_{i}\right)={\hat{f}}_{X,Y}\left(X,Y\right){\hat{f}}_{Y,Z}\left(Y,Z\right).$ The reason for estimating ϑ in this form is that, with the sample statistic $T_{n}^{\prime }\left(h\right)$, we can obtain a third order U-statistic representation of ϑ, similar to that for the DP test statistic, by which asymptotic normality follows.

The asymptotic normality of $T_{n}^{\prime }\left(h\right)$ is stated in Equation (3) below, which relies on the following two lemmas concerning the uniform consistency of density estimators.

**Lemma** **1.** *(Uniform consistency of $\hat{f}$) Let $\left\{W_{i}\right\}=\left\{\left(X_{i},Y_{i},Z_{i}\right)\right\},i\in N$ be a stationary sequence of 3-variate random variables with a continuous and bounded Lebesgue density f, satisfying the strong mixing conditions in Assumption 2 of [37]. If for the estimation of f, based on the first n values $W_{i}$, the kernel density estimator $f_{n}=\hat{f}$ is used with kernel function K(w), as given in Equation (6), with n-dependent bandwidth $h=h_{n}=\mathrm{cnst}.\times n^{-\beta }$, $\beta <\frac{1}{3}$, and K(w) is bounded and integrable, then*

$$\sup_{w\in R^{k}}\left|f_{n}\left(w\right)-f\left(w\right)\right|\to 0\;a.s.$$

**Proof.**  Equation (1) is a special case of Theorem 7 in [37], which more generally concerns the uniform consistency of the kernel estimator of *f* and its derivatives. □

Equation (1) provides the uniform consistency with probability one for a class of kernel estimators of multivariate density functions. This is a generalization of the consistency result of the univariate density estimation of [38,39] to the multivariate case with dependent observations. Note that to serve our purpose here we need uniform convergence, which is stronger than pointwise convergence. We refer to [40] for a detailed discussion between different types of convergence.

We next consider ${\tilde{T}}_{n}^{\prime }$, which differs from $T_{n}^{\prime }$ in Equation (14) only in having $\hat{v}\left(.\right)$ in the denominator replaced by the true unknown function v(.). In the next lemma, the short-hand notation $v_{i}=v\left(W_{i}\right)$ and $\eta_{i}=\eta \left(W_{i}\right)=f_{X,Y,Z}\left(X_{i},Y_{i},Z_{i}\right)f_{Y}\left(Y_{i}\right)-f_{X,Y}\left(X_{i},Y_{i}\right)f_{Y,Z}\left(Y_{i},Z_{i}\right)$ is used.

**Lemma** **2.** *Under the conditions of Lemma Equation (1), if in addition $\mathrm{Var}\left(\frac{\eta_{i}}{v_{i}}\right)<\infty$, then $\sqrt{n}\left(T_{n}^{\prime }\left(h_{n}\right)-\vartheta \right)$ and $\sqrt{n}\left({\tilde{T}}_{n}^{\prime }\left(h_{n}\right)-\vartheta \right)$ have the same limiting distribution. More formally stated,*

$$T_{n}^{\prime }\left(h_{n}\right)-{\tilde{T}}_{n}^{\prime }\left(h_{n}\right)=o_{P}\left(\frac{1}{\sqrt{n}}\right).$$

**Proof.**  See Equation (A.3). □

**Theorem** **3.** *If the bivariate time series ${\left\{\left(X_{t},Y_{t}\right)\right\}}_{t=1}^{n}$ is strictly stationary and satisfies at least one of the mixing conditions (a), (b) or (c) in Theorem 1 of [41], the corresponding random vector $\left(X_{t},Y_{t},Z_{t}\right)$ satisfies the conditions of Lemmas 1 and 2, and the density estimation kernel has bandwidth $h_{n}=Cn^{-\beta },C>0,\beta \in \left(\frac{1}{4},\frac{1}{3}\right)$, $T_{n}^{\prime }\left(h_{n}\right)$ is asymptotically normally distributed. In particular*

$$\sqrt{n}\frac{T_{n}^{\prime }\left(h_{n}\right)-\vartheta }{S_{n}}\overset{d}{⟶}N\left(0,1\right),$$

*where $S_{n}^{2}$ is an HAC estimator of the long-run variance $\sigma^{2}$ of $\sqrt{n}\left(T_{n}^{\prime }\left(h_{n}\right)-\vartheta \right)$.*

**Proof.**  See Equation (A.4). □

When implementing the test based on Equation (14), some comments regarding the treatment of the marginals are in order. Note that ϑ is invariant under invertible smooth transformations of the marginals due to the form of Equation (13) assuming that $X_{t}$ and $Y_{t}$ are continuous (the ratio of densities of the same variables is invariant under marginal transforms). Therefore, the dependence structure between $X_{t}$ and $Y_{t}$ remains intact under invertible marginal transforms. Although our testing framework does not depend crucially on the restrictive assumption of a uniform distribution for the time series as in [20,42], we recommend to use the probability integral transformation (PIT) on each of the marginals, as suggested by DP, as this usually improves the performance of statistical dependence tests. The reason is that, contrary to directly calculating the test statistics on the original data, the bounded support after transforming the marginals to a uniform distribution avoids non-existing moments during the bias and variance evaluation, which helps to stabilize the test statistic. There are alternative ways to transform the marginal variables into a bounded support, for example, by using a logistic function as [20]. Here, we decided to just apply the PIT, as it does not require any user-specified parameters, and always leads to identical (uniform) marginals. The procedure is to transform the original series $\left\{X_{t}\right\}$ ($\left\{Y_{t}\right\}$) to $\left\{U_{t}^{X}\right\}$ ($\left\{U_{t}^{Y}\right\}$) such that $\left\{U_{t}^{X}\right\}$ ($\left\{U_{t}^{Y}\right\}$) is the empirical CDF of $\left\{X_{t}\right\}$ ($\left\{Y_{t}\right\}$) and the empirical distribution of $\left\{U_{t}^{X}\right\}$ ($\left\{U_{t}^{Y}\right\}$) is uniform.

Since the transfer entropy-based measure ϑ is non-negative, tests based on the statistic $T_{n}^{\prime }\left(h\right)$ are implemented as one-sided tests, rejecting the null hypothesis if $\sqrt{n}T_{n}^{\prime }\left(h\right)/S_{n}>z_{1-\alpha }$, where $z_{1-\alpha }$ is the (1−α)th quantile of standard normal distribution for a given significance level α.

### 2.6. Bandwidth Selection

In nonparametric settings, there typically is no uniformly most powerful test against all alternatives. Hence, it is unlikely that a uniformly optimal bandwidth exists. As long as the bandwidth tends to zero with as $h=Cn^{-\beta },C>0,\beta \in \left(\frac{1}{4},\frac{1}{3}\right)$, our test has unit asymptotic power. Yet, we may define the optimal bandwidth in the sense of asymptotically minimal mean squared error (MSE). When balancing the first and forth leading terms in Equation (A5) to minimize the squared bias and variance, for a second order kernel, it is easy to show that the optimal bandwidth for the DP test is given by

(15) $$h_{\mathrm{DP}}=Cn^{-2/7},\;\mathrm{where}\;C={\left(\frac{13.5\;q_{2}}{{\left(E\left[s\left(W\right)\right]\right)}^{2}}\right)}^{1/7},$$

with $q_{2}$ and E[s(W)] the series expansion for the second moment of kernel function and expectation of bias, respectively. Since the convergence rate of the MSE, derived in Equation (A.4), is not affected by the way we construct the new test statistic, the derivation of Equation (15) remains intact and we calibrated the optimal bandwidth for our new test, finding

(16) $$h^{*}\approx 0.6h_{\mathrm{DP}},$$

where the scale factor 0.6 involved is a result of bias and variance adjustment for replacing the square kernel by the Gaussian kernel (the variance of the uniform DP kernel was $1/\sqrt{3}\approx 0.57735$, which we rounded off to 0.6). Intuitively, the $q_{2}$ and E[s(W)] terms are different from those for the DP test; more details can be found in Equation (A.5).

The optimal value for *C* is process-dependent and difficult to track analytically. For example, for a (G)ARCH process the optimal bandwidth is approximately given by $h_{DP}=Cn^{-2/7}$ where C≈8 (see DP). Applying Equation (16), we proceed with $h^{*}=4.8n^{-2/7}$ for (G)ARCH processes. To gain some insights into the bandwidth, we illustrate the test size and power with a 2-variate ARCH process, given by

(17) $$\begin{array}{cc} X_{t} & ∼N(0,1+aY_{t-1}^{2}),\\ Y_{t} & ∼N(0,1+aY_{t-1}^{2}). \end{array}$$

We let 0<a<0.4 and run 5000 Monte Carlo simulations for time series length varying from 200 to 5000. The size is assessed based on testing Granger non-causality from $\left\{X_{t}\right\}$ to $\left\{Y_{t}\right\}$, and for the power we use the same process but testing from Granger non-causality from $\left\{Y_{t}\right\}$ to $\left\{X_{t}\right\}$. The results are presented in Table 1, from which it can be seen that the modified DP test is conservative in the sense that its empirical size is lower than the nominal size 0.05 in all cases, while the power increases when *a* increases and when the sample size increases.

## 3. Size/Power Simulations

**Figure 3:**
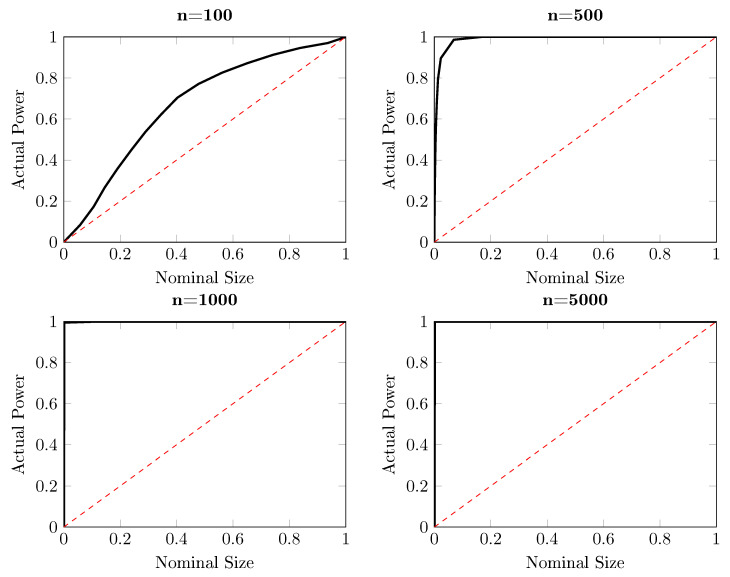
Size-power plots for the one-sided modified DP test for the artificial process $\left\{\left(X_{t},Y_{t},Z_{t}\right)\right\}$ for d=1/4, for which q=0, based on 10,000 independent replications. Each subplot shows the observed power against the nominal size for different sample sizes, ranging from 100 to 5000. The solid curve represents the observed power and the red dashed line corresponds with the diagonal, indicating the nominal size of the test.

This section investigates the performance of the modified DP test. Before proceeding with new data generating processes, we first revisit the example illustrated in Equation (9) for which the DP test fails to detect that $\left\{X_{t}\right\}$ is Granger causing $\left\{Y_{t}\right\}$. The modified DP test is performed with 10,000 replications, with the same bandwidth. The counterpart of the power-size plots for the DP test in Figure 2 is delivered in Figure 3. In contrast with the lack of power of the DP test, for time series length n=500 and larger, the modified DP test already has a very high power in this artificial experiment, as expected.

Next, we use numerical simulations to study the behavior of the modified DP test, while direct comparisons between the modified DP test $T_{n}^{\prime }$ and the DP test $T_{n}$ are also given. Three processes are being considered. In the first experiment, we consider a simple bivariate VAR process, given by

(18) $$\begin{array}{cccc} X_{t} & = & aY_{t-1}+\varepsilon_{x,t},\; & \;\varepsilon_{x,t}∼N\left(0,1\right),\\ Y_{t} & = & aY_{t-1}+\varepsilon_{y,t},\; & \;\varepsilon_{y,t}∼N\left(0,1\right). \end{array}$$

The second process is designed as a nonlinear VAR process in Equation (19). Again, the size and power are investigated by testing for Granger non-causality in two different directions.

(19) $$\begin{array}{cccc} X_{t} & = & 0.6X_{t-1}+aX_{t-1}Y_{t-1}+\varepsilon_{x,t}, & \;\varepsilon_{x,t}∼N\left(0,1\right),\\ Y_{t} & = & 0.6Y_{t-1}+\varepsilon_{y,t}, & \;\varepsilon_{y,t}∼N\left(0,1\right). \end{array}$$

The last process is the same as the example we used for illustrating the performance of the bandwidth selection rule, which is a bivariate ARCH process also given in Equation (17),

(20) $$\begin{array}{cc} X_{t} & ∼N(0,1+aY_{t-1}^{2}),\\ Y_{t} & ∼N(0,1+aY_{t-1}^{2}). \end{array}$$

**Figure 4:**
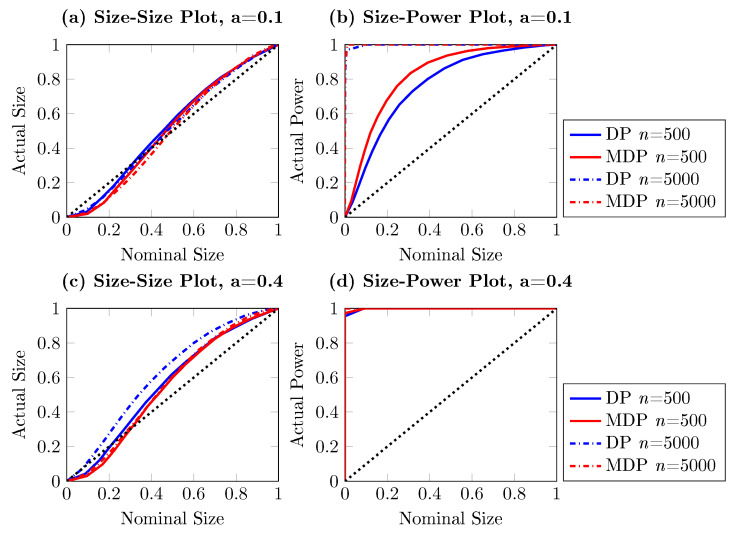
Size–size and size–power plots of Granger non-causality tests, based on 5000 simulations. The DGP is bivariate nonlinear vector autoregressive (VAR) as in Equation (19), with $\left\{Y_{t}\right\}$ Granger causing $\left\{X_{t}\right\}$. The left (right) column shows observed rejection rates under the null (alternative) hypothesis, the blue lines stand for DP test while the red lines indicate the modified DP test. The solid line and dashed line present results for sample size n=500 and n=5000, respectively.

**Figure 5:**
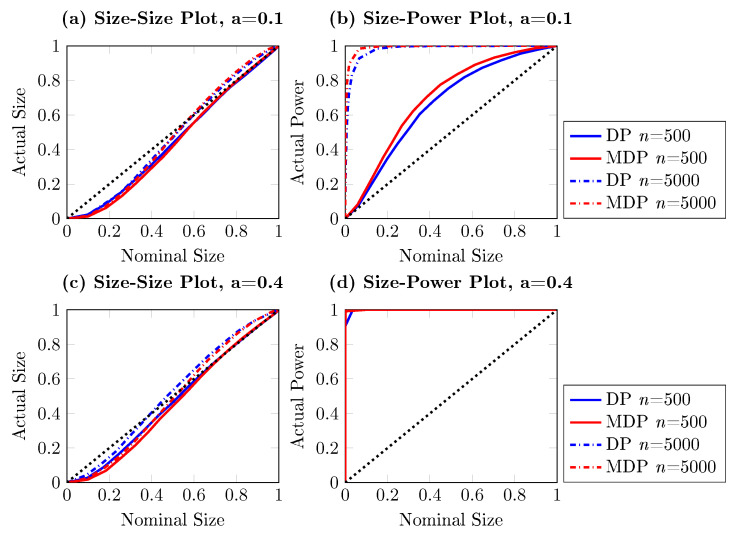
Size–size and size–power plots of Granger non-causality tests, based on 5000 simulations. The DGP is the bivariate linear VAR of Equation (18), with $\left\{Y_{t}\right\}$ Granger causing $\left\{X_{t}\right\}$. The left (right) column shows observed rejection rates under the null (alternative) hypothesis, the blue lines stand for the DP test while the red lines indicate the modified DP test. The solid line and dashed line present results for sample size n=500 and n=5000, respectively.

**Figure 6:**
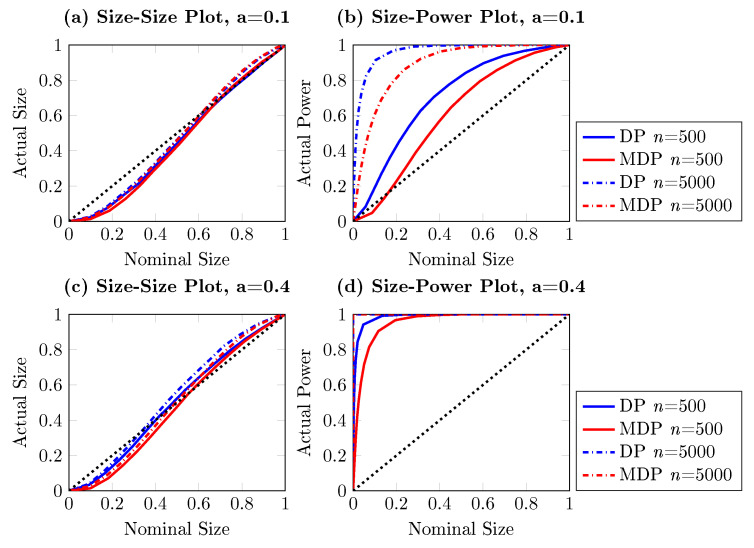
Size–size and size–power plots of Granger non-causality tests, based on 5000 simulations. The DGP is bivariate ARCH as in Equation (20), with $\left\{Y_{t}\right\}$ Granger causing $\left\{X_{t}\right\}$. The left (right) column shows observed rejection rates under the null (alternative) hypothesis, the blue lines stand for DP test while the red lines indicate the modified DP test. The solid line and dashed line present results for sample size n=500 and n=5000, respectively.

The results, which are shown in Figure 4, Figure 5 and Figure 6, are obtained with 5000 simulations for each process. We present the DP test and the modified DP test with both the empirical size–size and size–power plots for the three processes in Equations (18)–(20) for sample sizes n=500 and n=5000, respectively. The control parameter *a* is considered to take the values 0.1 and 0.4. As before, the empirical size is obtained by testing for Granger non-causality from $\left\{X_{t}\right\}$ to $\left\{Y_{t}\right\}$, and the empirical power is the observed rejection rate of testing for Granger non-causality from $\left\{Y_{t}\right\}$ to $\left\{X_{t}\right\}$.

It can be seen from Figure 4, Figure 5 and Figure 6 that the modified DP test is slightly more conservative than the DP test under the null hypothesis. However, the size distortion reduces when the sample size increases. The modified DP test is more powerful than the DP test in the linear and nonlinear VAR settings given in Equations (18) and (19). Overall, we see that the larger the sample size and the stronger the causal effect are, the better the asymptotic performance of the modified DP test is.

## 4. Empirical Illustration

### 4.1. Stock Volume–Return Relation

In this section, we first revisit the stock return–volume relation considered in [8] and DP. This topic has a long research history. Early empirical work mainly focused on the positive correlation between volume and stock price change, see [43]. The later literature exposed directional relations, for example, [44] found that large price movements are followed by high volume. In [45], authors observed a high-volume return premium; namely, periods of extremely high (low) volume tend to be followed by positive (negative) excess returns. More recently, [46] investigated the power law cross-correlations between price changes and volume changes of the S&P 500 Index over a long period.

We use daily volume and returns data for the three most-followed indices in US stock markets, the Standard and Poor’s 500 (S&P), the NASDAQ Composite (NASDAQ) and the Dow Jones Industrial Average (DJIA), between January 1985 and October 2016. The daily volume and adjusted daily closing prices were obtained from Yahoo Finance. The time series were converted by taking log returns multiplied by 100. In order to adjust for the day-of-the-week and month-of-the-year seasonal effects in both mean and variance of stock returns and volumes, we performed a two-stage adjustment process, similar to the procedure applied in [8]. We replace Akaike’s information criterion used by [8] with the [47] information criterion to be more stringent on picking up variables, having no intention to provoke a debate over the two criteria; we simply prefer a more parsimonious liner model to avoid potential overfitting. We apply our test not only to the raw data, but also on VAR filtered residuals and EGARCH(1,1,1) filtered residuals. We have tried different error distributions like normal, Students’ *t*, GED and Hansen’s skewed *t* [48]. The differences caused by different distributional assumptions are small; we only report the results based on the Students’ *t* distribution due to space considerations. The idea of filtering is to remove linear dependence and the effect of heteroskedasticity to isolate the nonlinear and higher moment relationships among series, respectively.

Table 2, Table 3 and Table 4 report the resulting *t* statistics for both the DP test and our modified DP test in both directions. The linear Granger F-values based on the optimal VAR models are also given. Two bandwidth values are used: 1.5 and 0.6, after standardization, where the latter value roughly corresponds to the derived optimal bandwidth (h=0.6138) and the larger bandwidth, also used in DP, is added as a robustness check.

Generally speaking, the results indicate that the effect in the return–volume direction is stronger than vice versa. For the test results on the raw data, the *F*-tests based on the linear VAR model and both nonparametric tests suggest evidence of return affecting volume for all three indexes. For the other direction, causality from volume to return, the linear Granger test finds no evidence of causal impact while the nonparametric tests claim strong causal effect except for the DJIA where only the modified DP test finds a causal link from volume to return. As argued above, the results for the linear test are suspicious since it only examines linear causal effects in the conditional mean; information exchange from higher moments is completely ignored.

A direct comparison between the DP test and the modified DP test shows that the new test is more powerful overall. For the unfiltered data, both tests find a strong causal effect in two directions for S&P and NASDAQ, but for the DJIA, the *t*-statistics of the DP test are weaker than those of the modified DP test. The bi-directional causality between return and volume remains unchanged after linear VAR filtration, although the DP test again shows weaker evidence. The result also suggests that the causality is strictly nonlinear. The linear test (*F*-test) is unable to spot these nonlinear linkages.

Further, in the direction from Volume to Return, these nonlinear causalities tend to vanish after EGARCH filtering. Thus, the bi-directional linkage is reduced to a one-directional relation from return to volume. The modified DP statistics, however, are in general larger than the DP *t*-values, and indicate more causal relations. In contrast with the DP test, our test suggests that the observed nonlinear causality cannot be completely attributed to second moment effects. Heteroskedasticity modeling may reduce this nonlinear feature to some extent, but its impact is not as strong as the DP test would suggest.

### 4.2. Application to Intraday Exchange Rates

In the second application, we apply the modified DP test to intraday exchange rates. We consider five major currencies: JPY, AUD, GBP, EUR and CHF, all against the USD. The data, obtained from Dukascopy Historical Data Feed, contain 5-min bid and ask quotes for the third quarter of 2016; from July 1 to September 30, with a total of 92 trading days and 26,496 high frequency observations. We use 5-minute data, corresponding to the sampling frequency of 288 quotes per day, which is high enough to avoid measurement errors (see [49]) but also low enough for the micro-structure not to be of major concern.

**Figure 7:**
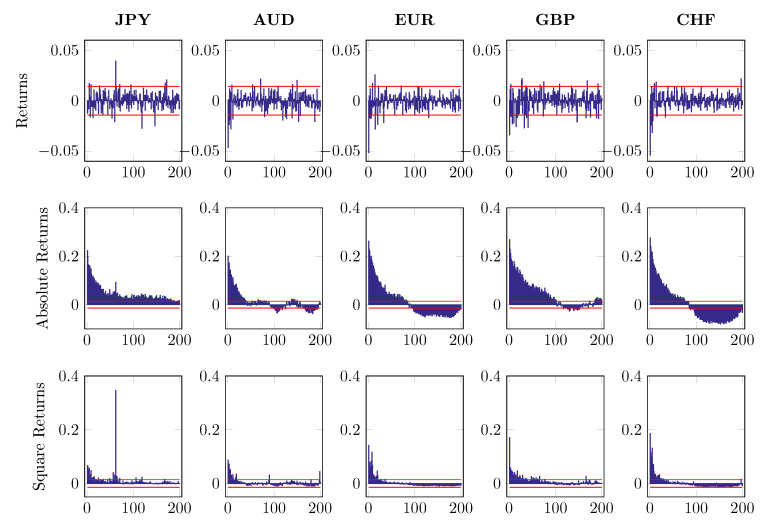
Autocorrelations of returns, absolute returns and square returns, up to 200 lags.

**Figure 8:**
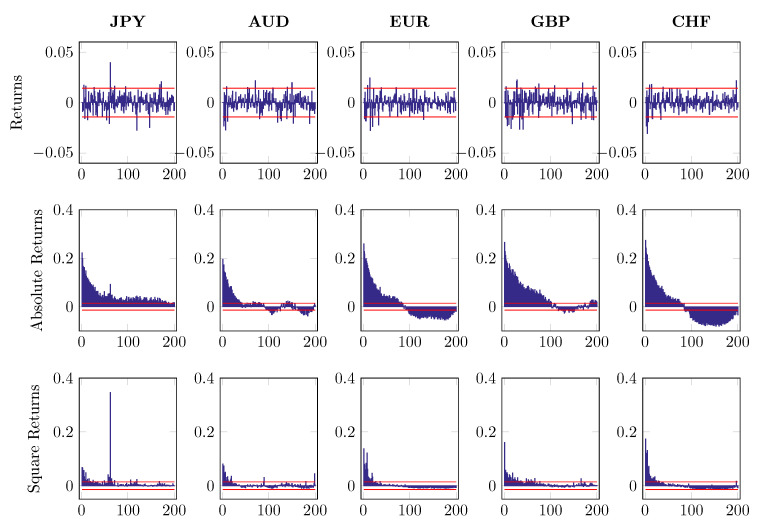
Autocorrelation of returns, absolute returns and square returns after MA(1) component is removed, up to 200 lags.

**Figure 9:**
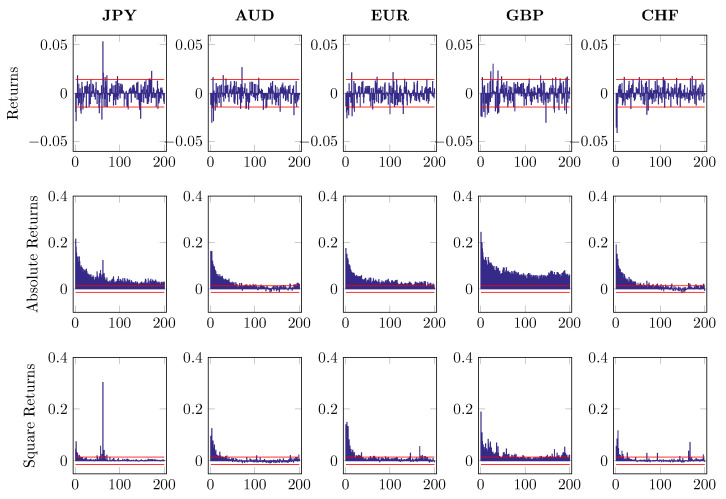
Autocorrelation of returns, absolute returns and square returns after MA(1) and GARCH filtering, up to 200 lags.

Although the foreign exchange market is one of the most active financial markets in the world, where trading takes place 24 h per day, intraday trading is not always active. Thus, we delete the thin trading period, from Friday 21:00 GMT until Sunday 20:55 GMT, also to keep the intraday periodicity intact. We calculate the exchange rate returns as in [50]. First, the average log bid and log ask prices are calculated, then the differences between the log prices at consecutive times are obtained. Next, we remove the conditional mean dynamics by fitting an MA(1) model and using the residuals as our return series following [51]. Finally, intraday seasonal effects are filtered out using estimated time-of-day dummies following [50], i.e.,

(21) $$r_{i,n,t}=d_{i,t}z_{i,n,t},$$

where $r_{i,n,t}$ denotes intraday log returns after MA(1) filtering. The subscript i=1,…,5 indicates five different currencies and n,t stands for time *t* on day *n*. The first component of return series $d_{i,t}$ refers to a deterministic intraday seasonal component while $z_{i,n,t}$ is the nonseasonal return portion, which is assumed to be independent of $d_{i,t}$. To distinguish $d_{i,t}$ from $z_{i,n,t}$, we fit the time-of-day dummies to $2\log|r_{i,n,t}|$ and use the estimated ${\hat{d}}_{i,t}$ to standardize the return $r_{i,n,t}$ with the restriction $\sum_{t=1}^{T}d_{i,t}=1$. Figure 7, Figure 8 and Figure 9 report the first 200 autocorrelations of returns, absolute returns and squared returns, when checking on the raw series, MA(1) residuals and EGARCH residuals, respectively.

We perform pairwise nonparametric Granger causality tests on the MA(1) filtered and seasonally adjusted data, as well as on the standardized residuals after EGARCH(1,1,1) filtering. We use the skewed *t* distribution introduced in [48] to model the innovation terms. We choose a bandwidth of 0.2768, according to Equation (16).

**Figure 10:**
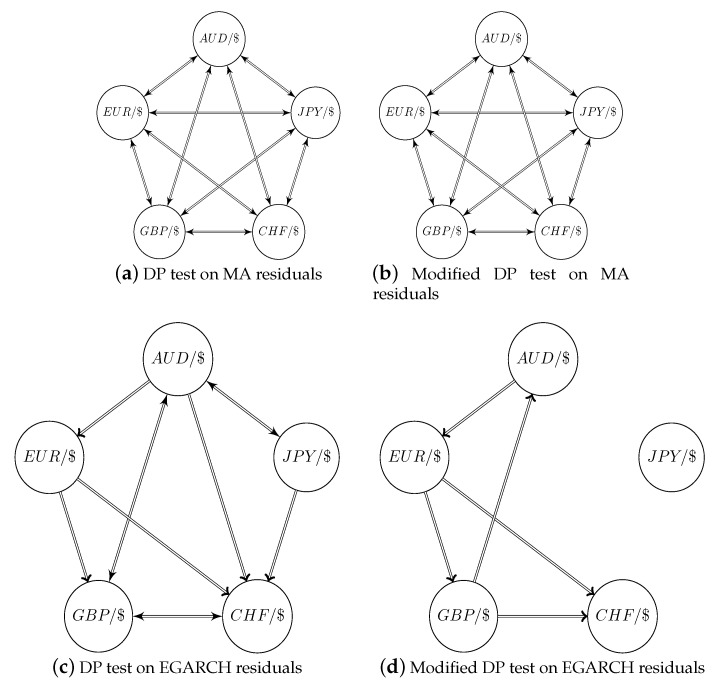
Graphical representation of pairwise causalities on MA and seasonally filtered residuals, as well as EGARCH filtered residuals. The arrows in the graph indicate a directional causality at the 5% level of significance.

The test results are shown in Table 5 for both MA(1) de-meaned and de-seasoned data, as well as EGARCH filtered data. Although not reported here, there is statistical evidence for strong bi-directional causality among all currency pairs on raw return data at 5-min lag. These bi-directional causalities do not disappear after removing the MA(1) component and seasonal component. However, the observed information spillover is significantly weaker after the EGARCH filtering. When testing based on the EGARCH standardized residuals, only a few pairs still show signs of a strong causal relation. Especially, the directional relation of EUR→CHF is the only one detected by both the DP test and the modified DP test at the 1% level of significance. A graphical representation is provided in Figure 10, where one can clearly see that most causal links are gone after EGARCH filtering. The modified DP test exposes five uni-directional linkages among the EGARCH filtered returns at the 5% level. The EUR and GBP are the most important driving currencies. While the DP test also admits the importance of JPY and particularly AUD, which shows bi-directional causality between JPY and GBP.

To sum up, we find evidence of strong causal links among exchange returns at an intraday high-frequency timescale. Each currency has predictive power for other currencies, implying high co-movements in the international exchange market. Although those directional linkages are not affected by the de-meaning procedure, we may reduce most of them by taking the volatility dynamics into account. When filtering out heteroskedasticity by EGARCH estimation, there only exist a few pairs containing spillover effects.

## 5. Summary and Conclusions

Borrowing the concept of transfer entropy from Information Theory, this paper develops a novel nonparametric test statistic for Granger non-causality. The asymptotic normality of the test statistic is derived by taking advantage of a U-statistic representation, similar to that applied in the DP test. The modified DP statistic, however, improves the DP statistic in at least the two respects: firstly, the positive definiteness of the quantity on which the test statistic is based, paves the way for properly testing for differences between conditional densities; secondly, the weight function in our test is motivated from an information-theoretical point of view, while the weight function in the DP test was selected in an ad hoc manner.

The simulation study confirms that the modified DP test has good size and power properties for a wide range of data generating processes. In the first application, a direct comparison with the DP test confirms that the DP test may suffer from a lack of power for specific processes, while the second application to high frequency exchange return data helps us better understand whether the spillover channel in exchange rate markets arises from conditional mean, conditional variance or higher conditional moments. Some obvious extensions to future work include the incorporation of additional lags of the variables and a generalization to higher-variate settings to allow for conditioning on additional, possibly confounding, variables.

## Figures and Tables

**Table 1:** Observed size and power of the $T_{n}^{\prime }\left(h\right)$ test for bivariate ARCH process Equation (17).

	*n*		200		500		1000		2000		5000
	*h*		1.0563		0.8130		0.6670		0.5471		0.4211
a=0.1	Size		0.0020		0.0016		0.0036		0.0016		0.0048
	Power		0.0032		0.0128		0.0288		0.0792		0.4000
a=0.2	Size		0.0020		0.0008		0.0032		0.0016		0.0048
	Power		0.0208		0.0932		0.2824		0.7292		0.9992
a=0.3	Size		0.0020		0.0012		0.0028		0.0032		0.0044
	Power		0.0816		0.3668		0.7916		0.9972		1.0000
a=0.4	Size		0.0020		0.0016		0.0032		0.0028		0.0084
	Power		0.1928		0.6968		0.9848		1.0000		1.0000

Note: Empirical size and power of the modified DP test for the process given in Equation (17) for different sample sizes and parameter *a*. The values represent observed rejection rates over 5000 realizations for nominal size 0.05.

**Table 2:** Test Statistics for the Standard and Poor’s 500 (S&P 500) returns and volume data. ‘MPD’ stands for ‘modified DP’.

	Volume → Return				
	Linear	DP		MDP	
		h=1.5	h=0.6	h=1.5	h=0.6
Raw data	0.8503	2.8769 **	2.9952 **	3.8526 **	2.7929 **
VAR residuals	-	3.6880 **	3.5683 **	4.2696 **	3.5769 **
EGARCH residuals	-	1.4403	1.2347	1.2672	2.4143 **
	Volume → Return				
	Linear	DP		MDP	
		h=1.5	h=0.6	h=1.5	h=0.6
Raw data	18.8302 **	4.9309 **	4.5138 **	4.9253 **	3.8359 **
VAR residuals	-	5.2732 **	5.1692 **	5.2239 **	3.7835 **
EGARCH residuals	-	3.0067 **	3.1214 **	3.1176 **	3.5101 **

Note: Test statistics for Granger causality between S&P 500 returns and volume data. Results for bandwidth values 1.5 and 0.6 are reported. Asterisks are used to indicate results that are significant at the 5% (${}^{*}$) and 1% (${}^{**}$) levels.

**Table 3:** Test Statistics for the NASDAQ returns and volume data.

	Volume → Return				
	Linear	DP		MDP	
		h=1.5	h=0.6	h=1.5	h=0.6
Raw data	0.0979	3.5894 **	3.3751 **	4.1311 **	3.3532 **
VAR residuals	-	4.3932 **	4.2931 **	5.3026 **	3.7300 **
EGARCH residuals	-	0.8282	0.5604	1.0430	1.2531
	Volume → Return				
	Linear	DP		MDP	
		h=1.5	h=0.6	h=1.5	h=0.6
Raw data	11.1736 **	5.1100 **	5.0201 **	4.9980 **	4.5935 **
VAR residuals	-	5.6293 **	6.4855 **	5.5750 **	5.0233 **
EGARCH residuals	-	3.5959 **	4.0693 **	4.0745 **	4.4522 **

Note: Test statistics for Granger causality between NASDAQ returns and volume data. Results for bandwidth values 1.5 and 0.6 are reported. Asterisks are used to indicate results that are significant at the 5% (${}^{*}$) and 1% (${}^{**}$) levels.

**Table 4:** Test Statistics for the Dow Jones Industrial Average (DJIA) returns and volume data. ’MDP’ stands for ‘modified DP’.

	Volume → Return				
	Linear	DP		MDP	
		h=1.5	h=0.6	h=1.5	h=0.6
Raw data	0.9761	1.2557	1.8384 *	1.9450 *	2.2697 *
VAR residuals	-	1.8711 *	2.0951 *	2.0998 *	2.7207 **
EGARCH residuals	-	1.4543	1.4317	1.0566	1.4801
	Volume → Return				
	Linear	DP		MDP	
		h=1.5	h=0.6	h=1.5	h=0.6
Raw data	17.0779 **	2.3076 *	1.7236 *	2.4972 **	3.3222 **
VAR residuals	-	2.3086 *	2.0454 *	2.6734 **	3.1056 **
EGARCH residuals	-	0.8033	1.0589	1.8989 *	3.0707 **

Note: Test statistics for the Granger causality between DJIA returns and volume data. Results for bandwidth values 1.5 and 0.6 are reported. Asterisks are used to indicate results that are significant at the 5% (${}^{*}$) and 1% (${}^{**}$) levels.

**Table 5:** Test statistics for the pairwise Granger causality on raw exchange returns.

Pair		MA Residuals		EGARCH Residuals	
		DP	MDP	DP	MDP
JPY	AUD	4.0180 **	3.0086 **	1.9843 *	1.2611
JPY	EUR	4.4724 **	3.7586 **	0.9336	0.5734
JPY	GBP	4.4096 **	3.9775 **	0.4305	0.3480
JPY	CHF	4.3236 **	3.9867 **	1.6542 *	1.5474
AUD	JPY	4.4872 **	3.6505 **	2.0162 *	1.4173
AUD	EUR	4.5398 **	3.5291 **	2.7414 **	1.9737 *
AUD	GBP	3.9936 **	2.8616 **	1.6532 *	0.6208
AUD	CHF	3.2458 **	3.2727 **	1.5546	1.5006
EUR	JPY	4.0257 **	3.2913 **	1.1139	0.3133
EUR	AUD	3.7456 **	3.1796 **	1.5543	1.0551
EUR	GBP	5.3053 **	4.3236 **	3.0613 **	2.2103 *
EUR	CHF	5.5101 **	4.6634 **	3.8299 **	3.5006 **
GBP	JPY	4.2506 **	3.4310 **	0.3216	0.0284
GBP	AUD	4.7248 **	4.0036 **	2.3418 **	1.9653 *
GBP	EUR	4.7092 **	3.9164 **	1.3648	0.5487
GBP	CHF	2.7094 **	2.2224 *	2.0109 *	1.6580 *
CHF	JPY	4.0033 **	3.4545 **	0.9972	0.3293
CHF	AUD	3.3506 **	2.5622 **	0.8823	0.0981
CHF	EUR	3.8227 **	2.6958 **	1.6378	0.2864
CHF	GBP	3.6522 **	3.0242 **	1.8381 *	1.5102

Note: Statistics for pairwise Granger non-causality tests on high-frequency returns of five major currencies. The data are first cleaned by the MA(1) and seasonal components, and then standardized based on the EGARCH conditional variance. Results are shown both for the DP test and the modified DP test with bandwidth h=0.2877. The asterisks indicate significance at the 5% (*) and 1% (**) levels.

## Appendix A

### Appendix A.1. Proof of Positive-Definiteness of the Transfer Entropy

According to the definition of TE in Equation (14), the expectation over the logarithm of the density ratio is evaluated: ${\mathrm{TE}}_{X\to Y}=E_{W}\left(\log\frac{f_{Z,X|Y}\left(Z,X|Y\right)}{f_{X|Y}\left(X|Y\right)f_{Z|Y}\left(Z|Y\right)}\right)$. Define the reciprocal of the density ratio in the logarithm as a random variable *R* in such a way: $R=\frac{f_{X|Y}\left(X|Y\right)f_{Z|Y}\left(Z|Y\right)}{f_{Z,X|Y}\left(Z,X|Y\right)}$, we could rewrite the TE as ${\mathrm{TE}}_{X\to Y}=E\left(-\log(R)\right)$. Since log(R) is a concave function of *R*, following Jensen’s inequality we have

(A1) $$E\left(\log(R)\right)\leq \log\left(E(R)\right).$$

Next, as random variable *R* is nonnegative since it is defined as a ratio of densities. For any realization of R=r>0, log(r)≤r−1. This is because as a concave function, log(r) is bounded from above by the tangent line at point (1,0), which is given by r−1. It follows that

(A2) $$\log\left(E(R)\right)\leq E\left(R\right)-1.$$

On combining Equations (A1) and (A2), we have $E\left(\log(R)\right)\leq E\left(R\right)-1=0$, where the last equality holds simply as a result of integral of the pdf over its full support delivering 1. A similar argument can be found in [52]. Thus, we have proved that ${\mathrm{TE}}_{X\to Y}\equiv -E\left(\log(R)\right)\geq 0$. It is obvious that the equality holds if and only if R=1, which is equivalent to $f_{Z,X|Y}\left(Z,X|Y\right)=f_{X|Y}\left(X|Y\right)f_{Z|Y}\left(Z|Y\right)$. This completes the proof of Equation (3).

### Appendix A.2. Proof of Positive-Definiteness of the First Order Te Statistic

Starting from Equation (11) and the definition of *t*,

(A3) $$\begin{array}{cc} \vartheta & =E_{W}\left(\frac{f_{Z,X|Y}\left(Z,X|Y\right)}{f_{X|Y}\left(X|Y\right)f_{Z|Y}\left(Z|Y\right)}-1\right)\\ \; & =\int \int \int \left(\frac{f_{Z,X|Y}\left(z,x|y\right)}{f_{X|Y}\left(x|y\right)f_{Z|Y}\left(z|y\right)}-1\right)f_{XYZ}\left(x,y,z\right)\;dx\;dy\;dz\\ \; & =\int \int \int \left(\frac{f_{Z,X|Y}^{2}\left(z,x|y\right)}{f_{X|Y}\left(x|y\right)f_{Z|Y}\left(z|y\right)}-f_{Z,X|Y}\left(z,x|y\right)\right)\;dx\;dz\;f_{Y}\left(y\right)\;dy\\ \; & =\int \int \int f_{X|Y}\left(x|y\right)f_{Z|Y}\left(z|y\right)\left(\frac{f_{Z,X|Y}^{2}\left(z,x|y\right)}{f_{X|Y}^{2}\left(x|y\right)f_{Z|Y}^{2}\left(z|y\right)}-\frac{f_{Z,X|Y}\left(z,x|y\right)}{f_{X|Y}\left(x|y\right)f_{Z|Y}\left(z|y\right)}\right)\;dx\;dz\;f_{Y}\left(y\right)\;dy\\ \; & =\int \int \int f_{X|Y}\left(x|y\right)f_{Z|Y}\left(z|y\right)\left(\frac{f_{Z,X|Y}^{2}\left(z,x|y\right)}{f_{X|Y}^{2}\left(x|y\right)f_{Z|Y}^{2}\left(z|y\right)}-2\frac{f_{Z,X|Y}\left(z,x|y\right)}{f_{X|Y}\left(x|y\right)f_{Z|Y}\left(z|y\right)}+1\right)\;dx\;dz\;f_{Y}\left(y\right)\;dy\\ \; & =\int \int \int f_{X|Y}\left(x|y\right)f_{Z|Y}\left(z|y\right){\left(\frac{f_{Z,X|Y}\left(z,x|y\right)}{f_{X|Y}\left(x|y\right)f_{Z|Y}\left(z|y\right)}-1\right)}^{2}\;dx\;dz\;f_{Y}\left(y\right)\;dy \end{array}$$

with equality if and only if $\frac{f_{Z,X|Y}\left(z,x|y\right)}{f_{X|Y}\left(x|y\right)f_{Z|Y}\left(z|y\right)}=1$ for any (x,y,z) in the support of (X,Y,Z). In the fourth step, we completed the square by using the fact that the integral over the whole support of the pdf is 1. Finally, ϑ≥0 follows naturally from the integrand being non-negative.

### Appendix A.3. Proof of Lemma 2

For a vector w=(x,y,z), let

$$\eta \left(w\right)=f_{X,Y,Z}\left(x,y,z\right)f_{Y}\left(y\right)-f_{X,Y}\left(x,y\right)f_{Y,Z}\left(y,x\right),$$

and

$$\hat{\eta }\left(w\right)={\hat{f}}_{X,Y,Z}\left(x,y,z\right){\hat{f}}_{Y}\left(y\right)-{\hat{f}}_{X,Y}\left(x,y\right){\hat{f}}_{Y,Z}\left(y,x\right).$$

By Equation (1), $\hat{f}\left(\cdot \right)\overset{a.s.}{⟶}f\left(\cdot \right)$ uniformly, as n→∞, but also $\hat{v}\left(\cdot \right)\overset{a.s.}{⟶}v\left(\cdot \right)$ and $\hat{\eta }\left(\cdot \right)\overset{a.s.}{⟶}\eta \left(\cdot \right)$ uniformly by the continuous mapping theorem.

Write $v_{i}=v\left(W_{i}\right)$ and $v_{i,n}=\hat{v}\left(W_{i}\right)$ for $v(W_{i})$ and its estimator based on $W_{1},\ldots ,W_{n}$, and likewise

$$\eta_{i}=\eta \left(W_{i}\right)$$

and

$$\eta_{i,n}={\hat{f}}_{X,Y,Z}\left(X_{i},Y_{i},Z_{i}\right){\hat{f}}_{Y}\left(Y_{i}\right)-{\hat{f}}_{X,Y}\left(X_{i},Y_{i}\right){\hat{f}}_{Y,Z}\left(Y_{i},Z_{i}\right).$$

One may write $T_{n}^{\prime }\left(h_{n}\right)=\frac{1}{n}\sum_{i=1}^{n}\frac{\eta_{i,n}}{v_{i,n}\left(h_{n}\right)}$ and ${\tilde{T}}_{n}^{\prime }\left(h_{n}\right)=\frac{1}{n}\sum_{i=1}^{n}\frac{\eta_{i,n}}{v_{i}}$. We then find that, up to higher-order terms in $n^{-1}$ resulting from the factor (n−1)/(n−2) in Equation (14),

$$\sqrt{n}\left(T_{n}^{\prime }\left(h_{n}\right)-{\tilde{T}}_{n}^{\prime }\left(h_{n}\right)\right)=\frac{1}{\sqrt{n}}\sum_{i=1}^{n}\frac{\eta_{i,n}}{v_{i}}\left(\frac{v_{i}}{v_{i,n}}-1\right).$$

The squared difference satisfies

$$\begin{array}{ccc} n{\left(T_{n}^{\prime }\left(h_{n}\right)-{\tilde{T}}_{n}^{\prime }\left(h_{n}\right)\right)}^{2}=n{\left(\frac{1}{n}\sum_{i=1}^{n}\frac{\eta_{i,n}}{v_{i}}\left(\frac{v_{i}}{v_{i,n}}-1\right)\right)}^{2} & \leq & \underset{j\in \{1,\ldots ,n\}}{\sup}\left({\left(\frac{v_{j}}{v_{j,n}}-1\right)}^{2}\right)\frac{1}{n}\sum_{i=1}^{n}{\left(\frac{\eta_{i,n}}{v_{i}}\right)}^{2}\\ & & \overset{P}{⟶}0\times E{\left(\left(\frac{\eta_{i}}{v_{i}}\right)\right)}^{2}. \end{array}$$

by the uniform convergence of $\hat{v}\left(\cdot \right)$. It follows that

$$T_{n}^{\prime }\left(h_{n}\right)-{\tilde{T}}_{n}^{\prime }\left(h_{n}\right)=o_{P}\left(\frac{1}{\sqrt{n}}\right),$$

if $\mathrm{Var}\left(\frac{\eta_{i}}{v_{i}}\right)<\infty$.

### Appendix A.4. Asymptotic Distribution of the Modified Dp Statistic

Applying Lemma 2, it remains to be proven that

$$\sqrt{n}\frac{\tilde{T_{n}^{\prime }}\left(h\right)-\vartheta }{S_{n}}\overset{d}{⟶}N\left(0,1\right).$$

According to the definition of $\tilde{T_{n}^{\prime }}\left(h\right)$, Equation (14) is a re-scaled DP statistic with the scaling factor (1/v(.)). In a similar manner of Theorem 1 in DP, we can obtain the asymptotic behavior of $\tilde{T_{n}^{\prime }}\left(h\right)$ by making use of the optimal mean squared error (MSE) bandwidth developed in [53] for this point estimator. For the moment, we consider the case where the vectors $W_{i}$ are assumed to be independent and identically distributed. We later allow for weak dependence in the time series context as described at the end of this section.

The test statistic $\tilde{T_{n}^{\prime }}\left(h\right)$ can be expressed by a degree three U-statistic $\tilde{K}\left(W_{i},W_{j},W_{k},h\right)$ by symmetrization with respect to the indices i,j,k. Further, defining two kernel functions as $\tilde{K_{1}}\left(w_{i}\right)=E\left[\tilde{K}\left(w_{i},W_{j},W_{k},h\right)\right]$ and $\tilde{K_{2}}\left(w_{i},w_{j},h\right)=E\left[\tilde{K}\left(w_{i},w_{j},W_{k},h\right)\right]$, we assume the three mild conditions adapting from [53] for controlling the rate of convergence of the point-wise bias as well as the serial expansions of the kernel functions, being

(A4) $$\begin{array}{cc} \tilde{K_{1}}\left(w_{i},h\right)-\lim_{h\to 0}\tilde{K_{1}}\left(w_{i},h\right) & =s\left(w_{i}\right)h^{\alpha }+s^{*}\left(w_{i},h\right),\;\alpha >0,\\ E[{\left(\tilde{K_{2}}\left(W_{i},W_{j},h\right)\right)}^{2}] & =q_{2}h^{-\gamma }+q_{2}^{*}\left(h\right),\;\gamma >0,\\ E[{\left(\tilde{K}\left(W_{i},W_{j},W_{k},h\right)\right)}^{2}] & =q_{3}h^{-\delta }+q_{3}^{*}\left(h\right),\;\delta >0, \end{array}$$

where all remainder terms are of higher orders, i.e., $E||s^{*}\left(W_{i},h\right){\left|\right|}^{2}=o_{P}\left(h^{2\alpha }\right)$, $q_{2}^{*}\left(h\right)=o_{P}\left(h^{-\gamma }\right)$ and $q_{3}^{*}\left(h\right)=o_{P}\left(h^{-\delta }\right)$ and the convergence rate is controlled by the parameters α, γ and δ. The conditions in Equation (A4) are satisfied if α is set as the order of kernel function K(.), which is 2 for the Gaussian kernel, and γ, δ depend on the dimensions of the variables under consideration via $\gamma =d_{X}+d_{Y}+d_{Z}$ and $\delta =d_{X}+2d_{Y}+d_{Z}$. Define $C_{0}=2\mathrm{Cov}\left(\lim_{h\to 0}\tilde{K_{1}}\left(W_{i},h\right),s\left(W_{i}\right)\right)$, we can show that the mean squared error of DP statistic as a function of sample size dependent bandwidth is given by

(A5) $$\begin{array}{c} \mathrm{MSE}\left[T_{n}\left(h\right)\right]={\left(E[s\left(W_{i}\right)]\right)}^{2}h^{2\alpha }+\frac{9}{n}C_{0}h^{\alpha }+\frac{9}{n}\mathrm{Var}\left[\lim_{h\to 0}\tilde{K_{1}}\left(W_{i},h\right)\right]+\frac{18}{n^{2}}q_{2}h^{-\gamma }+\frac{6}{n^{3}}q_{3}h^{-\delta }+h.o.t. \end{array}$$

endgroup.

The scaling factor (1/v(·)) in the modified test statistic $\tilde{T_{n}^{\prime }}\left(h\right)$ enters the MSE in Equation (A5) by mainly changing the bandwidth-independent variance term. For the other bandwidth-dependent terms, (1/v(·)) just re-scales the coefficients without affecting the convergence rates. Thus, we may still allow for all the *h*-dependent terms to be $o_{P}\left(n^{-1}\right)$ to ensure that $\frac{9}{n}\mathrm{Var}\left[\lim_{h\to 0}\tilde{K_{1}}\left(W_{i},h\right)\right]$-term asymptotically dominates (in which case asymptotic normality of the test statistic obtains). Therefore, adopting a sample size-dependent bandwidth $h_{n}=Cn^{-\beta }$, with *C*, β>0, one finds

(A6) $$\sqrt{n}\frac{\tilde{T_{n}^{\prime }}\left(h\right)-\vartheta }{S_{n}}\overset{d}{⟶}N\left(0,1\right)\;\mathrm{if}\;\frac{1}{2\alpha }<\beta <\frac{1}{d_{X}+d_{Y}+d_{Z}},$$

where $S_{n}^{2}$ is a consistent estimator of the asymptotic variance $9\mathrm{Var}\left[\lim_{h\to 0}\tilde{K_{1}}\left(W_{i},h\right)\right]$. The bivariate case, for α=2 and $d_{X}+d_{Y}+d_{Z}=3$, requires β∈(1/4,1/3). In the time series setting, under the assumption that the processes are stationary and satisfies at least one of the mixing conditions (a), (b) or (c) in Theorem 1 in [41], the long-run variance of $\sqrt{n}\left(T_{n}^{\prime }\left(h\right)-\vartheta \right)$ is given by $\sigma^{2}=9\left(\mathrm{Var}\left(U_{t}\right)+2\sum_{\ell =1}^{\infty }\mathrm{Cov}\left[U_{t},U_{t+\ell }\right]\right)$, where $U_{t}=\lim_{h\to 0}\left(\tilde{K_{1}}\left(W_{t},h\right)\right)$. The variance $\sigma^{2}$ can then be estimated using an HAC estimator for the long-run variance of $U_{t}$ [41,54].

### Appendix A.5. Optimal Bandwidth for the Modified Dp Test

The optimal bandwidth should balance the squared bias and variance of the test statistic, given in Equation (A5). Particularly, the first and fourth terms are leading, and all reminders are of higher order. The optimal bandwidth should have a similar form as the one for DP test in Equation (15).

In fact, the difference between two bandwidths is up to a scalar as a result of replacing the square kernel by a Gaussian one. Assuming the product kernel in Equation (6), the bias and variance of the density estimator are described by the following [24,55]:

(A7) $$\begin{array}{cc} \mathrm{Bias}(\hat{f}\left(x\right)) & =\frac{\mu_{\nu }\left(\kappa \right)}{\nu !}\sum_{j=1}^{k}\frac{\partial^{\nu }}{\partial x_{j}^{\nu }}f\left(x\right)h_{j}^{\nu }+o_{P}\left(h_{1}^{\nu }+\ldots +h_{k}^{\nu }\right),\\ \;\mathrm{Var}(\hat{f}\left(x\right)) & =\frac{f\left(x\right)R{\left(\kappa \right)}^{k}}{\left(n-1\right)h_{1}h_{2}\ldots h_{k}}+o_{P}\left(\left(n-1\right)h_{1}h_{2}\ldots h_{k}\right), \end{array}$$

where $\mu_{\nu }\left(\kappa \right)=\int_{-\infty }^{\infty }t^{\nu }\kappa \left(t\right)dt$ is the νth moment of a kernel function, with ν the corresponding order of the kernel. For Gaussian kernel κ(.), ν=2. The function $R\left(\kappa \right)=\int_{-\infty }^{\infty }\kappa {\left(t\right)}^{2}dt$ is the so called roughness function of the kernel. For a *k*-dimensional vector, the multivariate density estimation is carried out with a bandwidth vector $H={\left(h_{1},\ldots ,h_{k}\right)}^{\prime }$. It is not difficult to see that E[s(W)] and $q_{2}$ defined in Equation (A4) depend on the kernel function used trough the functions $\mu_{\nu }\left(\kappa \right)$ and R(κ).

Using the superscripts ‘G’ and ‘SQ’ to denote the Gaussian and square kernels respectively, [55] shows

(A8) $$\begin{array}{cc} \mu_{\nu }^{SQ}\left(\kappa \right) & =1/3,\;R^{SQ}\left(\kappa \right)=1/2,\\ \;\mu_{\nu }^{G}\left(\kappa \right) & =1,\;R^{G}\left(\kappa \right)=1/2\sqrt{\pi }. \end{array}$$

In our research, when we substitute the square kernel by the Gaussian kernel, the squared bias-related E[s(W)] and the variance-related $q_{2}$ will change correspondingly. Directly applying Equation (A8), we have $q^{G}{\left(\kappa \right)}_{2}=3q^{SQ}{\left(\kappa \right)}_{2}$, $R^{G}\left(\kappa \right)=R^{SQ}\left(\kappa \right)/\sqrt{\pi }$. After plugging this into Equation (15) and performing some calculations, one finds

(A9) $$h^{*}\approx 0.6h_{DP}.$$
